# The Potential Application of BAs for a Gas Sensor for Detecting SO_2_ Gas Molecule: a DFT Study

**DOI:** 10.1186/s11671-019-2972-4

**Published:** 2019-04-16

**Authors:** Jian Ren, Weijia Kong, Jiaming Ni

**Affiliations:** 1 School of Computer Science and Technology, Huaiyin Normal University, Chang Jiang West Road 111, Huaian, 223300 Jiangsu China; 2 Department of Chemistry, Beijing Normal University, No.19, Waidajie, Xinjiekou, Haidian District, Beijing, 100875 China; 3 School of Mechanical and Electrical Engineering, Guilin University of Electronic Technology, Jinji Road No.1, 54100 Gui, China

**Keywords:** Electronic structure, Density functional theory, Adsorption energy, Gas molecule, BAs

## Abstract

Different atmospheric gas molecules (e.g., N_2_, O_2_, CO_2_, H_2_O, CO, NO, NO_2_, NH_3_, and SO_2_) are absorbed on the pristine hexagonal boron arsenide (BAs) through density functional theory calculations. For each gas molecules, various adsorption positions were considered. The most stable adsorption depended on position, adsorption energy, charge transfer, and work function. SO_2_ gas molecules had the best adsorption energy, the shortest distance for BAs surface in the atmospheric gas molecule, and a certain amount of charge transfer. The calculation of work function was important for exploring the possibilities of adjusting the electronic and optical properties. Our results presented BAs materials can be the potential gas sensor of SO_2_ with high sensitivity and selectivity.

## Introduction

BAs (hexagonal boron arsenide) is composed of groups III and V elements. The groups of III–V elements have excellent properties, such as excellent photoelectric properties, mechanical properties, and large band gap [1]. The promising potential applications of 2D materials [2–5] were well documented in recent studies [6–20]; these materials had been used to recognize various biomolecules [21, 22], pollutants [23, 24], and gas molecules [25, 26] to develop suitable sensing devices. We had found more and more the groups of III–V element materials, for example, BN, AlN, GaN, GaAs, and BP, and it has more and more studies for the gas molecules by theoretical calculation. Strak et al. [27] discovered AlN(0001) was a powerful catalyst for high-pressure-high-temperature synthesis of ammonia, and the work also confirmed the possibility of the efficient synthesis of ammonia at the AlN(0001) surface. Diao et al. [28] presented adsorption of H_2_O, CO_2_, CO, H_2_, and N_2_ on (10–10) surfaces of pristine and Zn-doped GaAs nanowires; the effect of the adsorption of CO_2_ and N_2_ on absorption coefficients was the largest. Cheng et al. [29] showed the adsorption of most gas molecules on pure BP and doped BP by first principle study and concluded that N-BP was more suitable as a gas sensor for SO_2_, NO, and NO_2_ due to the existence of the desorption process. Kamaraj and Venkatesan [30] studied the structure and electronic properties of the BAs by the DFT and LDA; although considerable progress had been made in the experimental synthesis and theoretical study of BAs, the results of BAs nanosheets endowed the system with promising applications in nanoelectronics and photovoltaics.

In this work, we firstly investigated the gas sensing properties to fully exploit the possibilities of BAs as gas sensors by density functional theory (DFT) calculations. We predicted the adsorption properties of atmospheric gases (e.g., CO_2_, O_2_, N_2_, H_2_O, NO, NO_2_, NH_3_, CO, and SO_2_) on BAs based on first principle calculations. Our work demonstrated the apparent adsorption behavior, moderate charge transfers, and unique transmission characteristics of SO_2_ adsorption on BAs. The results suggested that monolayer BAs possessed great potential for SO_2_ sensing application.

## Theory and Method of Simulations

The system was modeled as a 4 × 4 supercell of BAs and atmospheric gas molecules adsorbed onto it. In DMol^3^ [31] calculation process, exchange-correlation factions were calculated within a general gradient approximate (GGA) with the Perdew-Burke-Ernzerhof (PBE) [32]. The Brillouin zone was sampled using a 5 × 5 × 1 Monkhorst-Pack k-point grid and Methfessel-Paxton smearing of 0.01 Ry. All the atomic structures were relaxed until the total energy and the Hellmann-Feynman force converged to 1.0 × 10^−5^ eV and 0.06 eV/Å [33].

To evaluate the interaction between gas molecules and adsorption sheet surface, we calculated the adsorption energy (*E*_ad_) of adsorbed systems, which was defined as:

$$ {E}_{\mathrm{ad}}={E}_{\mathrm{BAs}+\mathrm{gas}\mathrm{molecule}}-\left({E}_{\mathrm{BAs}}+{E}_{\mathrm{gas}\ \mathrm{molecule}}\right) $$

where *E*_BAs + gas molecule_ is the total energy of BAs-adsorbed system, *E*_BAs_ is the energy of BAs, and *E*_gas molecule_ is the energy of a gas molecule. All energies were calculated for optimized atomic structures. The charge transfer was investigated by Mulliken’s population analysis.

## Result and Discussion

**Fig. 1:**
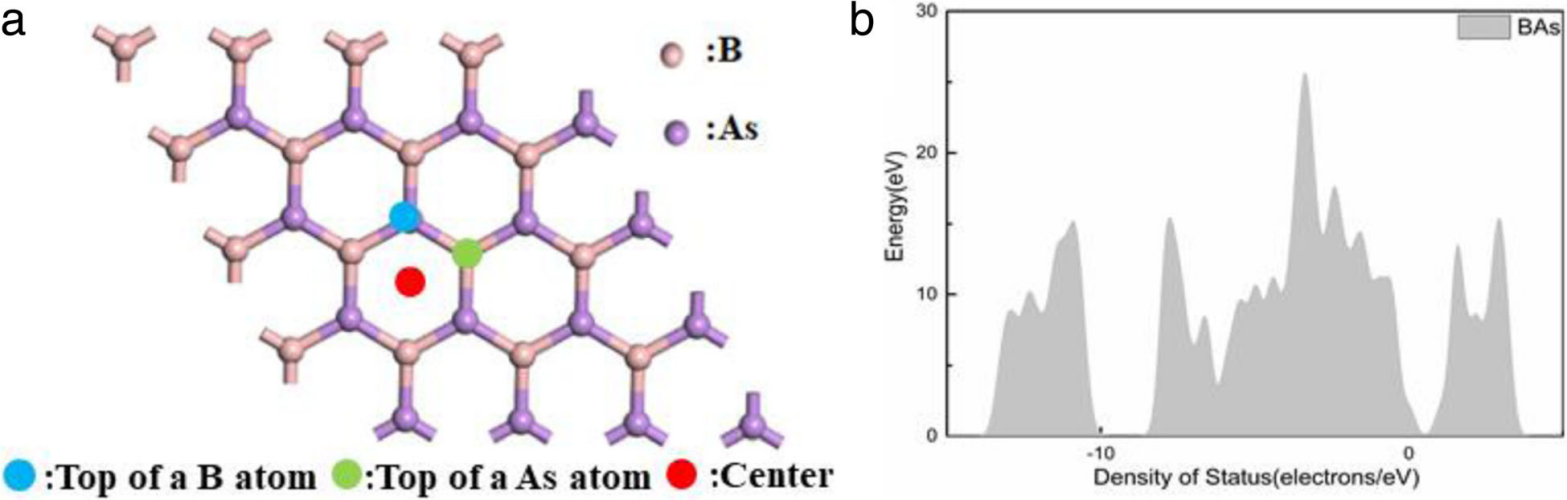
**a** Schematic view of top sites and center site on BAs. **b** The DOS of the BAs

Three adsorption sites were considered for BAs in this work, namely top of a boron atom (B), the top of an arsenic atom (As), and the center of a hexagonal B-As (center), as indicated in Fig. 1a. We studied the presence of the atmosphere and found the best gas sensor.

**Fig. 2:**
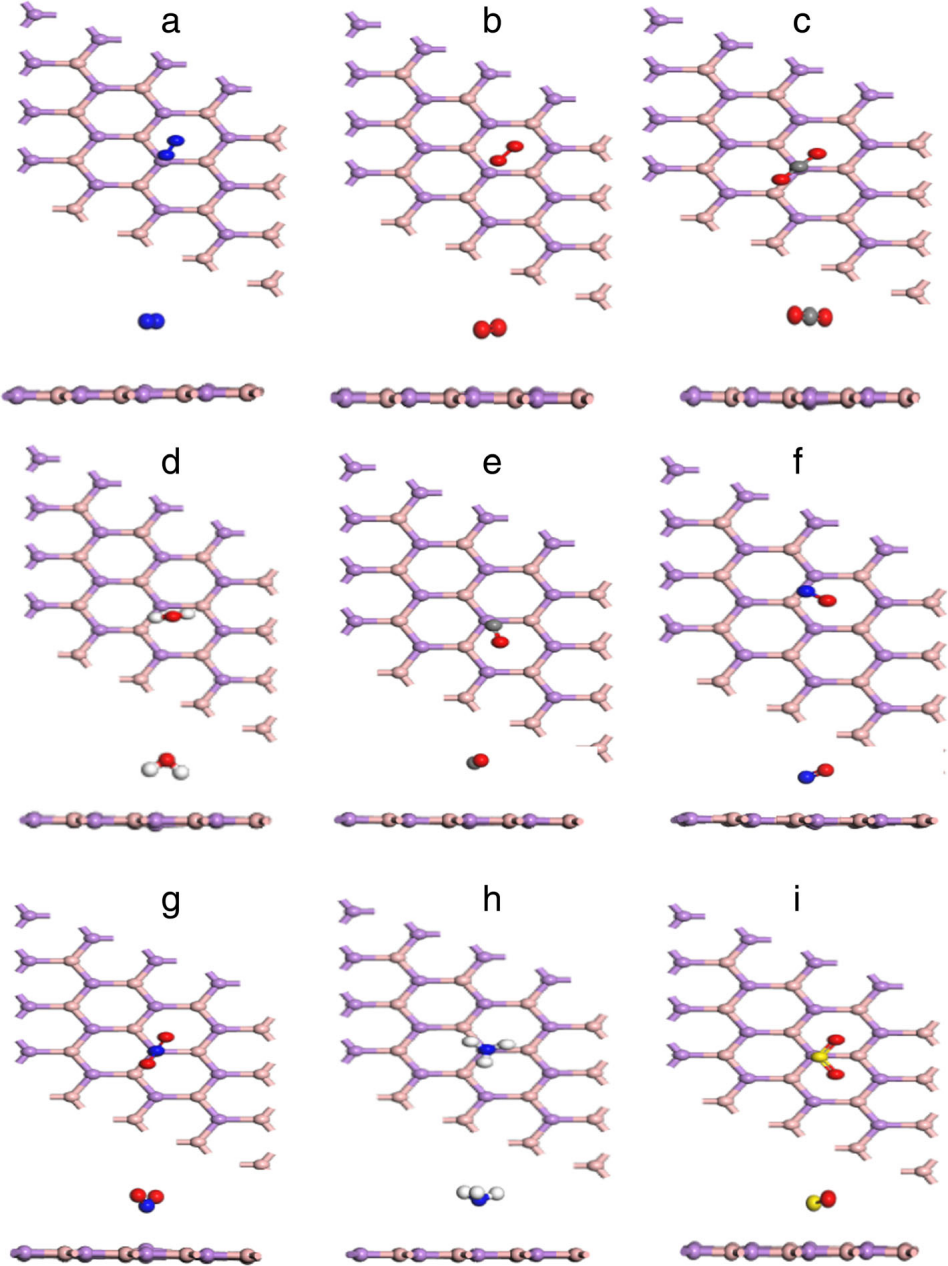
The most energetically favorable adsorption configurations of the gas molecules: N_2_ (**a**), O_2_ (**b**), CO_2_ (**c**), H_2_O (**d**), CO (**e**), NO (**f**), NO_2_ (**g**), NH_3_ (**h**), and SO_2_ (**i**) on monolayer BAs

First of all, the geometric structure of pristine BAs monolayer had been optimized, and as shown in Fig. 1b, BAs bond length was 1.967 Å. There was an indirect band gap of 1.381 eV to exhibit in the band structure of BAs sheet, which was smaller than that of the bulk structure. These values were in good agreement with the previously reported values (Fig. 2) [34, 35].

Meanwhile, we had analyzed the adsorption energy, the charge transfer, and the distance between the molecules and BAs surface. The final result was as shown in Table 1.

**Table 1:** Adsorption energy (*E*_ad_), Mulliken charge (*Q*) from the molecule to monolayer BAs, and distance (gas molecule/BAs) of the equilibrium nearest atom of gas molecule to atom of BAs monolayer

System	*E*_ad_ (eV)	*Q* (e)	*D* (Å)	Style
N_2_	− 0.24	− 0.014	3.65	Acceptor
O_2_	− 0.35	− 0.172	2.90	Acceptor
CO_2_	− 0.28	− 0.018	3.55	Acceptor
H_2_O	− 0.38	− 0.030	3.63	Acceptor
CO	− 0.27	− 0.024	3.50	Acceptor
NO	− 0.18	0.010	2.86	Donor
NO_2_	− 0.43	− 0.231	2.47	Acceptor
NH_3_	− 0.34	0.007	3.27	Donor
SO_2_	− 0.92	− 0.179	2.46	Acceptor

**Fig. 3:**
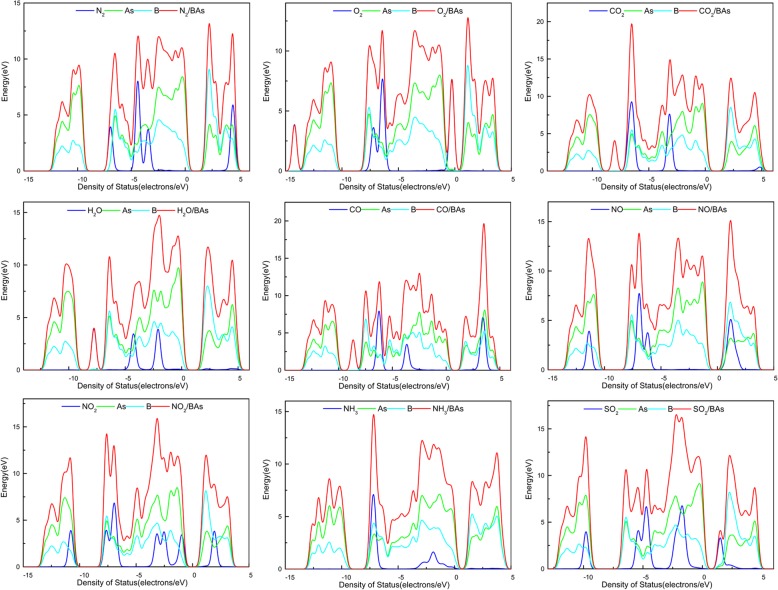
Density of states (DOS) of N_2_/BAs (**a**), O_2_/BAs (**b**), CO_2_/BAs (**c**), H_2_O/BAs (**d**), CO/BAs (**e**), NO/BAs (**f**), NO_2_/BAs (**g**), NH_3_/BAs (**h**) and SO_2_/BAs (**i**)

*N*_*2*_ *adsorption:* Adsorption of N_2_ gas molecule on BAs was studied for three configurations of N_2_/BAs, viz. top side of the B atom, top side of As atom, and the center of a hexagonal ring above the BAs surface, and the nearest distance was 3.764 Å, 3.549 Å, and 3.65 Å and corresponding adsorption energy was − 0.24 eV, − 0.27 eV, and − 0.24 eV, respectively. The center had the best adsorption energy and the most stable structure. The adsorption energy of N_2_BAs was − 0.24 eV, the charge transfer from BAs to N^2^ gas molecule was 0.014e, and the distance of the N2-BAs was 3.65 Å. Fig. 3a showed that there were many lines under the Fermi energy level, and the corresponding density of states had several peaks under the Fermi energy level. As shown in the figure, the N_2_ gas molecule had four peaks, which had a certain influence on BAs, mainly from − 5 to 0 eV, and had great contributions to the DOS. Overall, the effect of N_2_ gas molecule adsorption on BAs was poor.

*O*_*2*_ *adsorption:* O_2_ gas molecule tended to adsorb on the central point. The adsorption energy of O_2_/BAs was − 0.35 eV, and the distance of the O_2_-BAs was 2.90 Å. The total band structure and DOS for O_2_/BAs were plotted in Fig. 3. It was obvious that an extra line crossed the zero point and reduced the band gap; O_2_ gas molecule had a peak at − 1 to 0 eV and had an effect on the density of states above the Fermi level. The population analysis for Mulliken charge transfer showed that − 0.172e was transferred from BAs surface to the O_2_ gas molecule, suggesting that O_2_ gas molecule acted as an acceptor. In general, the O_2_ gas molecule adsorption on BAs was better than N_2_.

*CO*_*2*_ *adsorption:* CO_2_ gas molecule tended to adsorb on the top of As atom. The adsorption energy of CO^2^/BAs was − 0.28 eV, the charge transfer from BAs to CO^2^ gas molecule was − 0.018e, and the distance of the CO^2^-BAs was 3.55 Å. As shown in Fig. 3, compared to pristine BAs, the structure had no apparent change, and there were some obvious wave crests of the energy of − 9 eV in DOS, which had great contributions to the DOS. This point also highlighted the adsorption of CO_2_ gas molecule by BAs. The results showed that the adsorption effect and sensitivity of BAs to CO_2_ gas molecule were general.

*H*_*2*_*O adsorption:* H_2_O gas molecule tended to adsorb on the top of As atom. The adsorption energy of H^2^O/BAs was − 0.38 eV, the charge transfer from BAs to H^2^O gas molecule was − 0.03e, and the distance of the H^2^O-BAs was 3.63 Å. As shown in Fig. 3, there were no great changes in the structure compared to pristine BAs. The Fermi level of Al-G increased obviously and moved to the valence band. In general, the H_2_O gas molecule adsorption on BAs was ignored.

*CO adsorption:* CO gas molecule tended to adsorb on the top of As atom. The adsorption energy of CO/BAs was − 0.27 eV, the charge transfer from BAs to CO gas molecule was − 0.024e, and the distance of the CO-BAs was 3.50 Å. The total density of states (DOS) and band structure for BAs-CO were plotted in Fig. 3. CO gas molecule and As atom played a huge role in the effect of a peak of 3 to 4 eV on the DOS. However, there was no deviation in DOS in − 7 to 4 eV range, which suggested that CO was weekly adsorbed on BAs. There was some obvious wave crest of the energy of − 3 to 1 eV and 3 eV, which had great contributions to the DOS. The population analysis for Mulliken charge transfer showed that − 0.024e charge was transferred from BAs surface to the CO gas molecule, and it suggested that CO gas molecule acted as an acceptor. Overall, the effect of CO gas molecule adsorption on BAs was not special.

*NO adsorption:* NO gas molecule tended to adsorb on the top of B atom. The adsorption energy of NO/BAs was − 0.18 eV, the charge transfer was − 0.01e from NO gas molecule to BAs, and the distance of the NO-BAs was 2.86 Å. There were a lot of lines upon the Fermi energy level. It found that the energy gap in the middle band reduced the band gap value. From the density diagram of states, there was an extra wave peak above the Fermi energy level, but there was little change under the Fermi energy level, relatively stable in Fig. 3. The mixing of orbitals caused small charge transfer and redistribution over the interacting region. The population analysis for Mulliken charge transfer showed that 0.01e charge was transferred from BAs surface to the NO molecule, suggesting that NO acted as a donor. There was no deviation in DOS in − 7 to 4 eV range, which suggested that NO was weekly adsorbed on BAs.

*NO*_*2*_ *adsorption:* NO_2_ gas molecule tended to adsorb on the top of As atom. The adsorption energy of NO_2_/BAs was − 0.43 eV, and the distance of the NO_2_-BAs was 2.47 Å. The interesting was that the zero point in the band crossed a straight line directly after the adsorption of NO_2_ gas molecule, which meant that the BAs, which is a semiconductor, was transformed into the gold attribute; band gap was 0 eV. There was no great change in the whole, and a peak was generated at about − 3 eV due to NO_2_ gas molecular adsorption. There was some obvious wave crest of the energy of − 7 eV and 2 eV, which had great contributions to the DOS. In general, the adsorption of NO_2_ by BAs was better than that of several molecules above.

*NH*_*3*_ *adsorption:* NH_3_ gas molecule tended to adsorb on the top of As atom. The adsorption energy of NH^3^/ BA was -0.34 eV, the charge transfer from NH^3^ gas molecule to BA was 0.007e, and the distance of the NH^3^-BA was 3.27 Å. There was no clear change in the energy band and the density of states, except that there was an obvious peak of adsorption of NH_3_ gas molecule below the Fermi level. The NH_3_ gas molecule had a little impact on BAs at − 8 to − 4 eV, forming a 15 eV peak. The adsorption effect and sensitivity of its BAs to NH_3_ gas molecule were general.

*SO*_*2*_ *adsorption:* SO_2_ gas molecule tended to adsorb on the central point, the adsorption energy of SO_2_/BAs was − 0.92 eV, and the population analysis for Mulliken charge transfer showed that − 0.179e charge was transferred from BAs surface to the SO_2_ gas molecule, suggesting that SO_2_ gas molecule acts as an acceptor. The distance of the SO_2_/BAs was 2.46 Å. Compared to other gas molecules, the SO_2_/BAs had the biggest adsorption energy, the second largest electron transfer, and the shortest distance of the SO_2_-BAs. As shown in Fig. 3, the valence band of BAs had an obvious up and band gap decreased, and due to the adsorbed SO_2_ gas molecule, it could be seen from the density of states that there was one more wave peak at − 7.5 eV and certain transfer at the Fermi level. The adsorption of SO_2_ by BAs had the excellent effect.

**Fig. 4:**
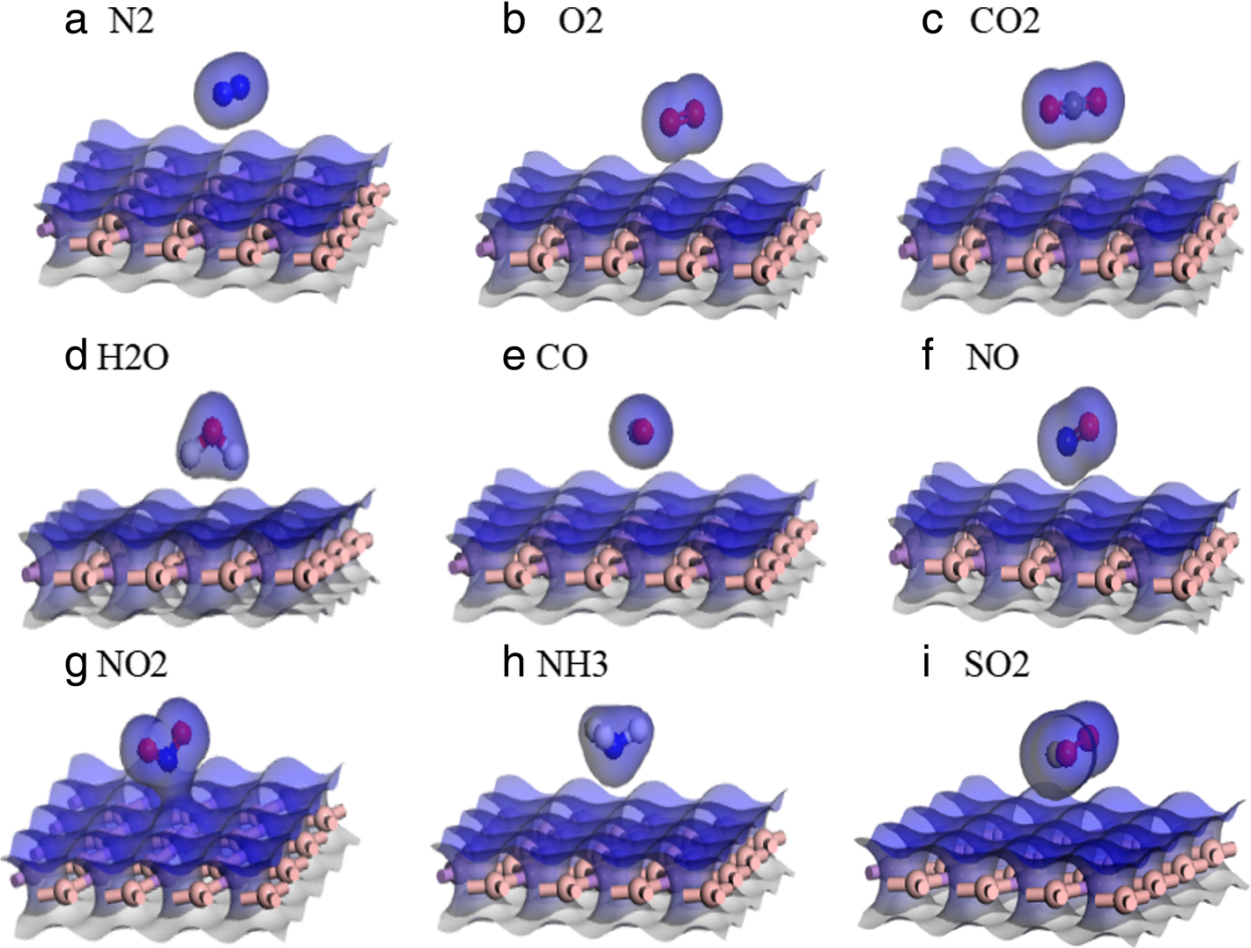
Electron density for pristine N_2_/BAs (**a**), O_2_/BAs (**b**), CO_2_/BAs (**c**), H_2_O/BAs (**d**), CO/BAs (**e**), NO/BAs (**f**), NO_2_/BAs (**g**), NH_3_/BAs (**h**), and SO_2_/BAs (**i**)

**Fig. 5:**
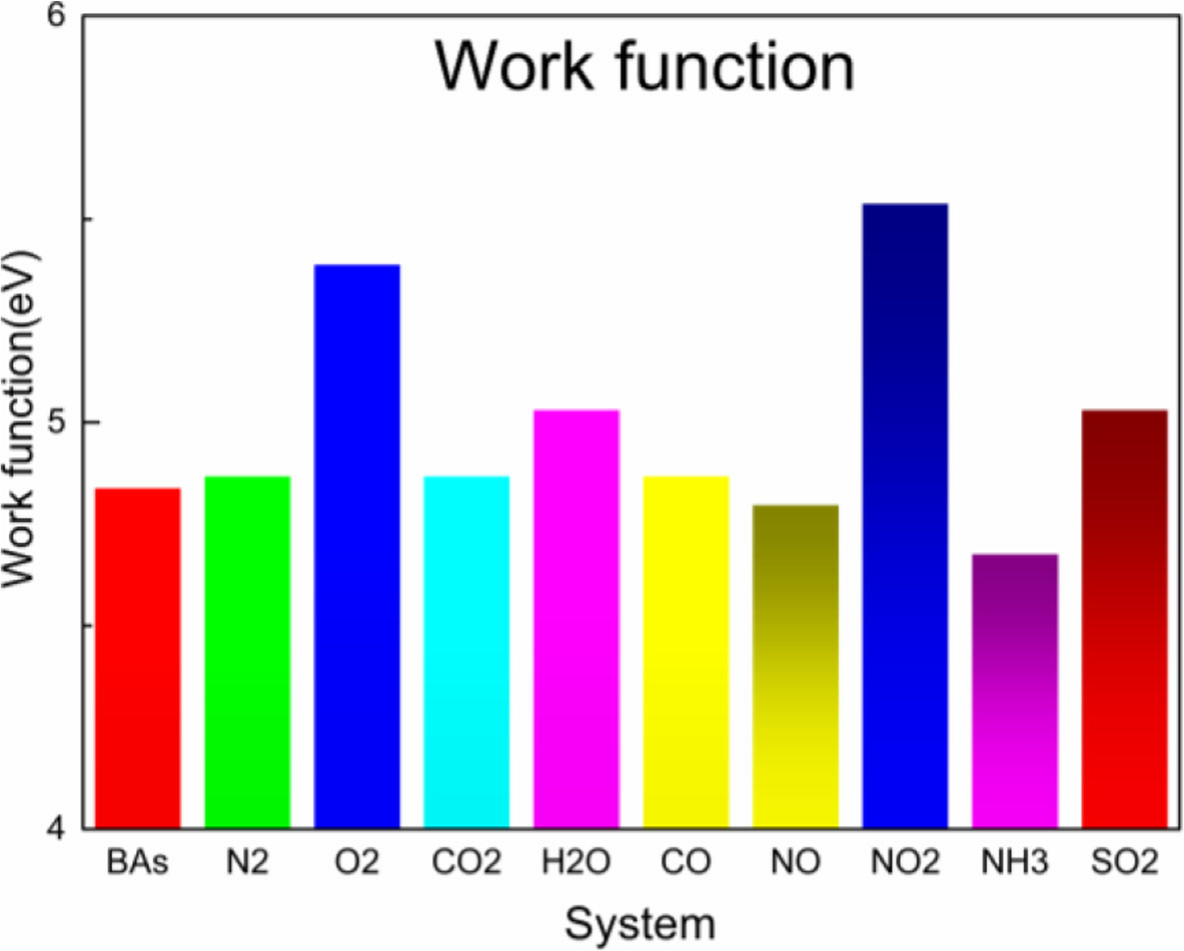
Work function of BAs N_2_/BAs, O_2_/BAs, CO_2_/BAs, H_2_O/BAs, CO/BAs, NO/BAs, NO_2_/BAs, NH_3_/BAs, and SO_2_/BAs

Fig. 4i showed the electron density diagram of SO_2_/BAs and the electron local overlap between BAs and SO_2_ gas molecule. On this basis, we drew the conclusion that the adsorption of SO_2_ by BAs was physical adsorption. The calculation of WF shown in Fig. 5 was of great significance in exploring the possibility of regulating the electronic and optical properties (such as absorption spectra and energy loss functions) by adsorbing small molecules. The work function was defined in solid physics as the minimum energy required to move an electron from the interior of a solid to the surface of the object. The work function of pristine BAs was 4.84 eV. NO and NH_3_ gas molecules were donors in charge transfer, and their work function decreased; the work function was 4.80 eV and 4.68 eV, respectively. The work function of N_2_/BAs, CO_2_/BAs, and CO/BAs was the same as that of BAs. The work function of O_2_/BAs, NO_2_/BAs, and SO_2_/BAs was higher than BAs. Combined with the above adsorption energy, distance of gas molecules and BAs surface, charge transfer, and work function, we found that SO_2_ gas molecule was most suitable for BAs materials.

## Conclusion

We have presented the structural and electronic properties of BAs with adsorbents N_2_, O_2_, CO_2_, H_2_O, CO, NO, NO_2_, NH_3_, and SO_2_ gas molecule, using density functional theory method. In the adsorption energy, SO_2_ > NO_2_ > H_2_O > O_2_ > NH_3_ > CO_2_ > CO > N_2_ > NO and SO_2_ < NO_2_ < NO<O_2_ < NH_3_ < CO < CO_2_ < H_2_O < N_2_ in the adsorption distance. NO_2_ has the largest *Q* and work function, maybe it could be detected by the proposed material because of good electrical response. SO_2_ gas molecule had the best adsorption energy, the shortest distance for gas molecule and BAs surface, and a certain amount of charge transfer. Combined with the above adsorption energy, distance of gas molecule and BAs surface, charge transfer, and work function, the current and the adsorption-induced current change of BAs exhibit strong anisotropic characteristics. Such sensitivity and selectivity to SO_2_ gas molecule adsorption make BAs a desirable candidate as a superior gas sensor.

## Abbreviations

BAs: Hexagonal boron arsenide
DOS: Density of states
WF: Work function
